# SUPPORTING DEMENTIA CAREGIVERS DURING COVID-19 WITH CAREHEROES IT: USAGE PATTERNS AND OUTCOMES

**DOI:** 10.1093/geroni/igac059.2792

**Published:** 2022-12-20

**Authors:** Nicole Ruggiano, Ellen Brown, C Victoria Framil, Shannon Hurley, Lisa Roberts, Peter Clarke, Sai Chaithra Allala, Jane Daquin

**Affiliations:** University of Alabama, Tuscaloosa, Alabama, United States; Florida International University, Miami, Florida, United States; Florida International University, Miami, Florida, United States; Florida State University, Tallahassee, Florida, United States; Florida International University, Miami, Florida, United States; Florida International University, Miami, Florida, United States; Florida International University, Miami, Florida, United States; University of Alabama, Tuscaloosa, Alabama, United States

## Abstract

Caregivers of people with dementia often experience negative physical and mental health outcomes due to the complex challenges posed by dementia symptoms and navigating care systems. The COVID-19 pandemic posed even greater hurdles for caregivers as many health care and support services transferred to virtual formats and required increased use of information technologies (IT). This research examined usage data and psychosocial outcomes of dementia caregivers who used a new mobile-enabled web-based app that was offered as an adjunct to clinical care at two memory clinics (Birmingham, AL; Miami, FL) during the pandemic. This app, called CareHeroes: (1) allows caregivers to self-assess their emotional and mental health; (2) offers a secure platform for tracking and communicating patient-related information among caregivers and providers; and (3) offers caregiver education. CareHeroes is also available in Spanish. Effort was made to recruit digitally underserved populations of caregivers (e.g., rural dwelling, underserved racial/ethnic groups). This presentation will review caregiver outcomes (e.g., burden, depression, user data) at baseline and 3-month follow-up. Among the 22 caregivers who enrolled in the study, 17 completed baseline and 3-month follow-up interviews. Over a 14-month period, participants logged onto CareHeroes 131 times, which varied by month. Caregivers most often used CareHeroes to complete assessment tools. Findings indicate that depression and burden were lower at 3-month follow-up compared to baseline, though this reduction was not statistically significant. The presentation will also review challenges of integrating a new technology intervention designed to promote telehealth during the COVID-19 pandemic.

